# Adaptive Morley element algorithms for the biharmonic eigenvalue problem

**DOI:** 10.1186/s13660-018-1643-9

**Published:** 2018-03-06

**Authors:** Hao Li, Yidu Yang

**Affiliations:** The School of the Mathematical Sciences, Guizhou Normal University, Gui Yang, China

**Keywords:** 65N25, 65N30, 65N15, Biharmonic eigenvalues, Morley elements, Adaptive algorithms, An inequality on Rayleigh quotient

## Abstract

This paper is devoted to the adaptive Morley element algorithms for a biharmonic eigenvalue problem in $\mathbb{R}^{n}$ ($n\geq2$). We combine the Morley element method with the shifted-inverse iteration including Rayleigh quotient iteration and the inverse iteration with fixed shift to propose multigrid discretization schemes in an adaptive fashion. We establish an inequality on Rayleigh quotient and use it to prove the efficiency of the adaptive algorithms. Numerical experiments show that these algorithms are efficient and can get the optimal convergence rate.

## Introduction

Biharmonic equation/eigenvalue problem plays an important role in elastic mechanics. In 1968, Morley designed a famous non-conforming element called the Morley element [1] to solve biharmonic equation (plate bending problem). The Morley element was extended to arbitrarily dimensions by Wang and Xu [2] in 2006. For biharmonic equation, the a priori/a posteriori error estimate was studied in [3–6] and the convergence and optimality of the adaptive Morley element method was proved in [7, 8]. The Morley element has been employed to solve the biharmonic eigenvalue problem, including the vibration of a plate; and [9] studied its a priori error estimate. [10, 11] studied a posteriori error estimate and the adaptive method, [12] adopted a new method dispensing with any additional regularity assumption to study the error estimates and adaptive algorithms. This paper further studies the adaptive Morley element method and has the following features:

1. The adaptive finite element methods, which were first proposed by Babuska and Rheinboldt [13], have gained an extensive attention in academia. More and more researchers entered this field and obtained many good results, most of which have been systemically summarized in [5, 14–16]. And [10, 12] have employed the adaptive Morley element algorithms for the biharmonic eigenvalue problem based on solving directly the original eigenvalue problem $a(u,v)=\lambda b(u,v)$ in each iteration. In this paper, we establish the adaptive Morley element algorithms based on the shifted-inverse iteration including Rayleigh quotient iteration and the inverse iteration with fixed shift to solve the biharmonic eigenvalue problem. The shifted-inverse iteration method based on the multigrid discretizations has been studied in-depth (see [17] and the references therein), but they did not involve the Morley element. With our method, the solution of an original eigenvalue problem is reduced to the solution of an eigenvalue problem on a much coarser grid and the solution of a series of linear algebraic equations on finer and finer grids. Therefore, our method is more efficient than the method in [10, 12].
2. For fourth order equations in $\mathbb{R}^{3}$, it is difficult to employ a conforming element. For instance, Zenicek constructed a conforming tetrahedral finite element with 9 degree of polynomials and 220 nodal parameters [5], while the Morley tetrahedral element [2] has only 10 nodal parameters. Based on [4], we comply with the adaptive Morley element computation for the biharmonic eigenvalue problem in $\mathbb{R}^{3}$. Numerical results indicate that the adaptive algorithms are very efficient.
3. A family of good adaptive meshes should satisfy $h=O(h_{\min}^{\alpha})$, where *h* is the mesh size, $h_{\min}$ is the diameter of the smallest element, and *α* is the regularity index of the biharmonic equation over the domain with reentrant corner (see [18]). However, we find through the numerical computation that $\frac{h}{h_{\min}^{\alpha}}$ will become bigger and bigger when the iteration increases for the standard adaptive algorithm. Thus, referring to [19], we combine the standard local refined adaptive algorithm with uniformly refined algorithm to give new algorithms.

## Preliminary

Consider the following biharmonic eigenvalue problem:

2.1 $$ \begin{aligned} &\Delta^{2}u=\lambda u,\quad \mbox{in } \Omega, \\ &\frac{\partial u}{\partial\gamma}=0,\quad u=0,\mbox{ on }\partial\Omega, \end{aligned} $$

where $\Omega\in \mathbb{R}^{n}$ is a polyhedral domain with boundary *∂*Ω, $\frac{\partial u}{\partial\gamma}$ is the outward normal derivative on *∂*Ω.

Let $H^{s}(\Omega)$ denote a usual Sobolev space with norm $\Vert \cdot \Vert _{s,\Omega}$ ($\Vert \cdot \Vert _{s}$), $H_{0}^{2}(\Omega)=\{v\in H^{2}(\Omega): v|_{\partial\Omega}=\frac{\partial v}{\partial \gamma}|_{\partial\Omega}=0\}$ with norm $\Vert \cdot \Vert _{2}$ and semi-norm $|\cdot|_{2}$.

The weak form of (2.1) is to seek $(\lambda,u)\in \mathbb{R}\times H_{0}^{2}(\Omega)$ with $u\neq 0$ such that

2.2 $$ a(u,v)=\lambda b(u,v),\quad \forall v\in H_{0}^{2}( \Omega), $$

where

$$\begin{aligned} a(u,v)= \int_{\Omega}\sum_{1\leq i,j\leq n} \frac{\partial^{2} u}{\partial x_{i}\partial x_{j}}\frac{\partial^{2} v}{\partial x_{i}\partial x_{j}}\,dx,\qquad b(u,v)= \int_{\Omega}uv \,dx,\qquad \Vert u \Vert _{b}= \sqrt{b(u,u)}. \end{aligned}$$

In the case of $n=2$, (2.2) is the weak form of clamped plate vibration.

It is easy to verify that $a(u,v)$ is a symmetric, continuous, and $H_{0}^{2}(\Omega)$-elliptic bilinear form. Let $\Vert u \Vert _{a}=\sqrt{a(u,u)}$, then the norms $\Vert u \Vert _{a}$, $\Vert u \Vert _{2}$, and $|u|_{2}$ are equivalent.

We assume that $\pi_{h}=\{\kappa\}$ is a regular simplex partition of Ω and satisfies $\overline{\Omega}=\bigcup\overline{\kappa}$ (see [20]). Let $h_{\kappa}$ be the diameter of *κ*, and $h=\max\{h_{\kappa}: \kappa\in \pi_{h}\}$ be the mesh size of $\pi_{h}$ ($h<1$), $h_{\min}=\min\{h_{\kappa}: \kappa\in \pi_{h}\}$. Let $\varepsilon_{h}=\{F\}$ denote the set of faces (($n-1$)-simplexes) of $\pi_{h}$, and let $\varepsilon_{h}'=\{l\}$ denote the set of faces ($n-2$)-simplexes of $\pi_{h}$. When $n=2$, $l=z$ is a vertex of *κ*, and $\frac{1}{\operatorname{meas}(l)}\int_{l}v=v(z)$. Let $\pi_{h}(\kappa)$ denote the set of all elements sharing a common face with the element *κ*. Let $\kappa_{+}$ and $\kappa_{-}$ be any two *n*-simplexes with a face *F* in common such that the unit outward normal to $\kappa_{-}$ at *F* corresponds to $\gamma_{F}$. We denote the jump of *v* across the face *F* by

$$[v]=(v|_{\kappa_{+}}-v|_{\kappa_{-}})|_{F}. $$

And the jump on boundary faces is simply given by the trace of the function on each face.

In the papers [2, 5], the Morley element space is defined by

$$\begin{aligned} S^{h} =& \biggl\{ v\in L_{2}(\Omega):v\mid_{\kappa} \in P_{2}(\kappa),\forall \kappa\in \pi_{h}, \int_{F}[\nabla v\cdot \gamma_{F}]=0\ \forall F\in \varepsilon_{h},\\ & \frac{1}{\operatorname{meas}(l)} \int_{l}[v]=0\ \forall l\in \varepsilon_{h}' \biggr\} , \end{aligned}$$

where $P_{2}(\kappa)$ denotes the space of polynomials of degree less than or equal to 2 on *κ*.

Define the interpolation operator $I_{h}:H_{0}^{2}(\Omega)\rightarrow S^{h}$, which satisfies

$$\begin{aligned} \int_{F}\frac{\partial I_{h} v}{\partial\gamma}= \int_{F}\frac{\partial v}{\partial\gamma}\quad \forall F\in \varepsilon_{h},\qquad \frac{1}{l} \int_{l} I_{h}v=\frac{1}{l} \int_{l} v\quad \forall l\in\varepsilon_{h}'. \end{aligned}$$

The Morley element space $S^{h}\subset L_{2}(\Omega)$, $S^{h}\not\subset H^{1}(\Omega)$. Let

$$\begin{aligned} \Vert v \Vert _{m,h}^{2}=\sum _{\kappa\in \pi_{h}} \Vert v \Vert _{m,\kappa}^{2},\qquad \vert v \vert _{m,h}^{2}=\sum _{\kappa\in \pi_{h}} \vert v \vert _{m,\kappa}^{2},\quad m=0,1,2. \end{aligned}$$

From Lemma 8 in [2], we know that $|\cdot|_{2,h}$ is equivalent to $\Vert \cdot \Vert _{2,h}$, $\Vert \cdot \Vert _{2,h}$ is a norm in $S^{h}$, and $a_{h}(\cdot,\cdot)$ is a uniformly $S^{h}$-elliptic bilinear form, and $\Vert \cdot \Vert _{h}=a_{h}(\cdot,\cdot)^{\frac{1}{2}}$ is a norm in $S^{h}$. And the following equality holds for any $w\in H_{0}^{2}(\Omega)$:

$$\lim_{h\rightarrow0}\inf_{v\in S^{h}} \Vert w-v \Vert _{h}=0. $$

The discrete form of (2.2) reads: Find $(\lambda_{h},u_{h})\in \mathbb{R}\times S^{h}$ with $u_{h}\neq0$ such that

2.3 $$\begin{aligned} a_{h}(u_{h},v)=\lambda_{h} b(u_{h},v),\quad \forall v\in S^{h}, \end{aligned}$$

where

$$\begin{aligned} a_{h}(u_{h},v)= \sum_{\kappa\in\pi_{h}} \int_{\kappa} \sum_{i,j=1}^{n} \frac{\partial^{2}u_{h}}{\partial x_{i}\partial x_{j}}\frac{\partial^{2}v}{\partial x_{i}\partial x_{j}}\,dx. \end{aligned}$$

The corresponding boundary value problem of (2.1) is

2.4 $$\begin{aligned} &\Delta^{2}w=f,\quad \mbox{in }\Omega, \\ &\frac{\partial w}{\partial\gamma}=0,\quad w=0,\mbox{ on }\partial\Omega. \end{aligned}$$

From [18], we know that

$$\Vert w \Vert _{2+\alpha}\lesssim \Vert f \Vert _{0}, $$

where $\alpha\in(\frac{1}{2},1)$ for the domain with reentrant corner, and $\alpha=1$ for the convex domain in $\mathbb{R}^{2}$.

The weak form of (2.4) and its discrete form are to find $w\in H_{0}^{2}(\Omega)$ such that

$$a(w,v)=b(f,v),\quad \forall v\in H_{0}^{2}(\Omega), $$

and to find $w_{h}\in S^{h}$ such that

$$a_{h}(w_{h},v)=b(f,v),\quad \forall v\in S^{h}. $$

Define the solution operators $T:L_{2}({\Omega})\to H_{0}^{2}(\Omega)\subset L_{2}({\Omega})$ and $T_{h}: L_{2}({\Omega})\to S^{h}$ as follows:

2.5 $$ \begin{aligned} &a(Tf,v)=b(f,v),\quad \forall v \in H_{0}^{2}( \Omega), \\ &a_{h}(T_{h}f,v)=b(f,v),\quad \forall v \in S^{h}. \end{aligned} $$

Then $T, T_{h}: L_{2}(\Omega) \to L_{2}(\Omega)$ are self-adjoint and compact.

It is well known that the eigenvalue problem (2.1) has countably many eigenvalues, which are real and positive diverging to +∞. Suppose that *λ* and $\lambda_{h}$ are the *k*th eigenvalue of (2.2) and (2.3), respectively, the algebraic multiplicity of *λ* is equal to *q*, $\lambda=\lambda_{k}=\lambda_{k+1}=\cdots=\lambda_{k+q-1}$. Let $M(\lambda)$ be the space spanned by all eigenfunctions corresponding to *λ* and $M_{h}(\lambda)$ be the direct sum of eigenspaces corresponding to all eigenvalues of (2.3) that converge to *λ*. Let $\hat{M}(\lambda)=\{u:u\in M(\lambda), \Vert u \Vert _{h}=1\}$.

Now we introduce the following quantity:

2.6 $$ \delta_{h}(\lambda)= \bigl\Vert (T-T_{h})|_{M(\lambda_{k})} \bigr\Vert _{h}. $$

The saturation condition was analyzed in [21–23], especially, it was analyzed in [22] for very general cases. According to this condition, we can make the following assumption:

2.7 $$ C_{1}h\leq \inf_{\forall v\in S^{h}} \Vert u-v \Vert _{h}\leq C_{2}\delta_{h}( \lambda_{k}),\quad \forall u\in M(\lambda_{k}), $$

where $C_{1}$ and $C_{2}$ are independent of mesh parameters.

Define $S^{h}+H_{0}^{2}(\Omega)=\{v_{h}+v: v_{h}\in S^{h}, v\in H_{0}^{2}(\Omega)\}$.

Due to the generalized Poincare–Friedrichs inequality, Theorem 3 in [24] and $a_{h}(u-I_{h}u,v)=0, \forall v\in S^{h}$ (see [5]), we deduce for any $w\in S^{h}, u\in H_{0}^{2}(\Omega)$

$$\begin{aligned} \Vert w-u \Vert _{0} \leq& \Vert w-I_{h}u \Vert _{0}+ \Vert u-I_{h}u \Vert _{0} \\ \leq& \frac{C_{3}}{3} \bigl( \Vert w-I_{h}u \Vert _{h}+h^{2} \Vert u-I_{h}u \Vert _{h} \bigr) \\ \leq& \frac{C_{3}}{3} \bigl( \Vert w-u \Vert _{h}+ \Vert u-I_{h}u \Vert _{h}+h^{2} \Vert u-I_{h}u \Vert _{h} \bigr) \leq C_{3} \Vert w-u \Vert _{h}. \end{aligned}$$

Therefore,

2.8 $$\begin{aligned} \Vert v \Vert _{0}\leq C_{3} \Vert v \Vert _{h},\quad \forall v\in S^{h}+H_{0}^{2}( \Omega), \end{aligned}$$

where $C_{3}$ is a positive constant independent of mesh parameters.

From (2.5) we have the following estimate using the Cauchy–Schwarz inequality: For any $g\in L^{2}(\Omega), T_{h} g\in S^{h}$ satisfying

$$\Vert T_{h} g \Vert _{h}\leq C_{3} \Vert g \Vert _{0}, $$

define the consistency term

$$E_{h}(w,v_{h})=a_{h}(w,v)-b(f,v),\quad \forall v\in S^{h}+H_{0}^{2}(\Omega). $$

Suppose $w\in H^{2+r}(\Omega)$, $r\in (\frac{1}{2},1 ]$, then we have the following estimate:

2.9 $$ \bigl\vert E_{h}(w,v) \bigr\vert \leq C_{4} h^{r} \bigl( \Vert w \Vert _{2+r}+h^{2-r} \Vert f \Vert _{0} \bigr) \Vert v \Vert _{h}, \quad \forall v\in S^{h}+H_{0}^{2}(\Omega). $$

Using the trace inequality [5] proves the above estimate under the case $r=1$. Using the arguments in [5], we can obtain the above estimate under the case $r=(\frac{1}{2},1]$ (also see [11]).

We can derive the following Lemma 2.1 from Lemma 2.3 in [25].

### Lemma 2.1

*Let* *λ* *and* $\lambda_{h}$ *be the* $kth$ *eigenvalue of* (2.2) *and* (2.3), *respectively*. *Then for any eigenfunction* $u_{h}$ *corresponding to* $\lambda_{h}$ *with* $\Vert u_{h} \Vert _{h}=1$, *there exist* $u\in M(\lambda)$ *and* $h_{0}>0$ *such that if* $h\leq h_{0}$,

2.10 $$ \Vert u-u_{h} \Vert _{h}\leq C_{5}\delta_{h}(\lambda), $$

*for any* $u\in\widehat{M}(\lambda)$, *there exists* $u_{h}\in M_{h}(\lambda)$ *such that if* $h\leq h_{0}$,

2.11 $$ \Vert u-u_{h} \Vert _{h}\leq C_{6}\delta_{h}(\lambda), $$

*where constants* $C_{5}$ *and* $C_{6}$ *are positive and only depend on* *λ*.

The following inequality on Rayleigh quotient plays an important role.

### Theorem 2.1

*Let* $(\lambda,u)$ *be an eigenpair of* (2.2), $v\in S^{h}$ *with* $\Vert v \Vert _{h}=1$ *and* $\Vert v-u \Vert _{h}\leq (4 C_{3}\sqrt{\lambda})^{-1}$, *then the Rayleigh quotient* $R(v)=\frac{a_{h}(v,v)}{ \Vert v \Vert ^{2}_{0}}$ *satisfies*

2.12 $$ \bigl\vert R(v)-\lambda \bigr\vert \leq C_{7} \Vert v-u \Vert _{h}^{1+r}, $$

*where* $C_{7}=4\lambda (1+\lambda C_{3}^{2} )(4C_{3}\sqrt{\lambda})^{r-1}+\frac{8C_{4}}{C_{1}^{r}}\lambda ( \Vert u \Vert _{2+r}+h^{2-r}\lambda \Vert u \Vert _{0} )$.

### Proof

Since $u\in M(\lambda), v\in S^{h}, \Vert v \Vert _{h}=1$ and $\Vert v-u \Vert _{h}\leq (4 C_{3}\sqrt{\lambda})^{-1}$, by Lemma 3.1 in [26] we have

$$\begin{aligned} & \biggl\Vert v-\frac{u}{ \Vert u \Vert _{h}} \biggr\Vert _{h}\leq 2 \Vert v-u \Vert _{h}\leq (2 C_{3}\sqrt{\lambda})^{-1}, \\ & \biggl\Vert v-\frac{u}{ \Vert u \Vert _{h}} \biggr\Vert _{0}\leq C_{3} \biggl\Vert v-\frac{u}{ \Vert u \Vert _{h}} \biggr\Vert _{h} \leq \frac{1}{2\sqrt{\lambda}}, \end{aligned}$$

which together with $\Vert \frac{u}{ \Vert u \Vert _{h}} \Vert _{0}=\frac{1}{\sqrt{\lambda}}$ yields

$$\Vert v \Vert _{0}\geq \biggl\Vert \frac{u}{ \Vert u \Vert _{h}} \biggr\Vert _{0}- \biggl\Vert v-\frac{u}{ \Vert u \Vert _{h}} \biggr\Vert _{0}\geq\frac{1}{2\sqrt{\lambda}}. $$

By Lemma 2.5 in [26], we get

$$\frac{a_{h}(v,v)}{ \Vert v \Vert ^{2}_{0}}-\lambda=\frac{ \Vert v-u \Vert _{h}^{2}}{ \Vert v \Vert _{0}^{2}}-\lambda\frac{ \Vert v-u \Vert _{0}^{2}}{ \Vert v \Vert _{0}^{2}}+2 \frac{E_{h}(u,v)}{ \Vert v \Vert _{0}^{2}}. $$

Hence, from inequalities (2.7)–(2.9) we deduce

$$\begin{aligned} \bigl\vert R(v)-\lambda \bigr\vert &\leq4\lambda \Vert v-u \Vert _{h}^{2}+4\lambda^{2} \Vert v-u \Vert _{0}^{2}+8\lambda E_{h}(u,v) \\ &\leq4\lambda \Vert v-u \Vert _{h}^{2}+4C_{3}^{2} \lambda^{2} \Vert v-u \Vert _{h}^{2}+8\lambda E_{h}(u,v-u) \\ &\leq4\lambda \bigl(1+\lambda C_{3}^{2} \bigr) \Vert v-u \Vert _{h}^{2}+8C_{4}h^{r}\lambda \bigl( \Vert u \Vert _{2+r}+h^{2-r}\lambda \Vert u \Vert _{0} \bigr) \Vert v-u \Vert _{h} \\ &\leq4\lambda \bigl(1+\lambda C_{3}^{2} \bigr) \Vert v-u \Vert _{h}^{2}+8C_{4}\lambda \bigl( \Vert u \Vert _{2+r}+h^{2-r}\lambda \Vert u \Vert _{0} \bigr) \Vert v-u \Vert _{h}^{1+r} \\ &\leq \biggl(4\lambda \bigl(1+\lambda C_{3}^{2} \bigr) \Vert v-u \Vert _{h}^{1-r}+\frac{8C_{4}}{C_{1}^{r}}\lambda \bigl( \Vert u \Vert _{2+r}+h^{2-r}\lambda \Vert u \Vert _{0} \bigr) \biggr) \Vert v-u \Vert _{h}^{1+r} \\ &\leq \biggl(4\lambda \bigl(1+\lambda C_{3}^{2} \bigr) (4C_{3}\sqrt{\lambda})^{r-1}+\frac{8C_{4}}{C_{1}^{r}}\lambda \bigl( \Vert u \Vert _{2+r}+h^{2-r}\lambda \Vert u \Vert _{0} \bigr) \biggr) \Vert v-u \Vert _{h}^{1+r} \\ &\leq C_{7} \Vert v-u \Vert _{h}^{1+r}. \end{aligned}$$

We get the results that we need. □

(2.3) implies $\lambda_{h}=R(u_{h})$, and from (2.7), (2.10), and (3.22) in [11], we deduce

2.13 $$ \bigl\vert R(u_{h})-\lambda \bigr\vert \leq C_{7} \Vert u_{h}-u \Vert _{h}^{2} \leq C_{5}^{2}C_{7}\delta_{h}^{2}( \lambda). $$

## The shifted-inverse iteration based on multigrid discretization

Let $\{S^{h_{i}}\}_{0}^{\infty}$ be a family Morley element spaces, $h_{0}=H$. Refer to the references [17], we present the following calculation schemes.

### Scheme 1

(Rayleigh quotient iteration based on multigrid discretizations)

Given the iteration times *l*.

*Step 1.* Solve (2.3) on $S^{H}$: Find $(\lambda_{H},u_{H})\in \mathbb{R}\times S^{H}$ such that $\Vert u_{H} \Vert _{H}=1$ and

$$a_{H}(u_{H},v)=\lambda_{H}b(u_{H},v), \quad \forall v\in S^{H}. $$

*Step 2.* $u^{h_{0}}\Leftarrow u_{H}, \lambda^{h_{0}}\Leftarrow \lambda_{H}, i\Leftarrow 1$.

*Step 3.* Solve a linear system on $S^{h_{i}}$: Find $u'\in S^{h_{i}}$ such that

$$a_{h} \bigl(u',v \bigr)-\lambda^{h_{i-1}}b \bigl(u',v \bigr)=b \bigl(u^{h_{i-1}},v \bigr), \quad \forall v \in S^{h_{i}}, $$

set $u^{h_{i}}=\frac{u'}{ \Vert u' \Vert _{h}}$.

*Step 4.* Compute the Rayleigh quotient:

$$\lambda^{h_{i}}=\frac{a_{h}(u^{h_{i}},u^{h_{i}})}{b(u^{h_{i}},u^{h_{i}})}. $$

*Step 5.* If $i=l$, then output $(\lambda^{h_{l}},u^{h_{l}})$, stop; else, $i\Leftarrow i+1$, and return to Step 3.

### Scheme 2

(The inverse iteration with fixed shift based on multigrid discretizations)

Given the iteration times *l* and $i_{0}$.

*Steps 1*∼*4.* The same as Steps 1–4 in Scheme 1.

*Step 5.* If $i>i_{0}$, then $\lambda^{h_{i_{0}}}\Leftarrow \lambda^{h_{i-1}}$, $i\Leftarrow i+1$, turn to Step 6; else, $i\Leftarrow i+1$, and return to Step 3.

*Step 6.* Solve a linear system on $S^{h_{i}}$: Find $u'\in S ^{h_{i}}$ such that

$$a_{h} \bigl(u',v \bigr)-\lambda^{h_{i_{0}}}b \bigl(u',v \bigr)=b \bigl(u^{h_{i-1}},v \bigr),\quad \forall v \in S^{h_{i}}, $$

set $u^{h_{i}}=\frac{u'}{ \Vert u' \Vert _{h}}$.

*Step 7.* Compute the Rayleigh quotient

$$\lambda^{h_{i}}=\frac{a_{h}(u^{h_{i}},u^{h_{i}})}{b(u^{h_{i}},u^{h_{i}})}. $$

*Step 8.* If $i=l$, then output $(\lambda^{h_{l}},u^{h_{l}})$, stop; else, $i\Leftarrow i+1$, and return to Step 6.

Strictly speaking, the above $a_{h}(\cdot,\cdot)$ and $\Vert \cdot \Vert _{h}$ should be written as $a_{h_{i}}(\cdot,\cdot)$ and $\Vert \cdot \Vert _{h_{i}}$. For the sake of simplicity, we write $a_{h_{i}}(\cdot,\cdot)$ and $\Vert \cdot \Vert _{h_{i}}$ as $a_{h}(\cdot,\cdot)$ and $\Vert \cdot \Vert _{h}$, in this paper.

## The theoretical analysis

In this section, we will prove the convergence of $(\lambda^{h_{l}},u^{h_{l}})$ derived from Scheme 1/Scheme 2, and that the constants appearing in the error estimates are not only independent of mesh parameter but also iterative times *l*.

In the following discussion, let $(\lambda_{k},u_{k})$ and $(\lambda_{k,h},u_{k,h})$ denote the $kth$ eigenpair of (2.2) and (2.3), respectively, and $\mu_{k}=\frac{1}{\lambda_{k}},\mu_{k,h}=\frac{1}{\lambda_{k,h}},M(\mu_{k})=M(\lambda_{k}),M_{h}(\mu_{k})=M_{h}(\lambda_{k})$.

Denote $\operatorname{dist}(u,S)=\inf_{v\in S} \Vert u-v \Vert _{h}$.

Our analysis is based on the following Lemma 4.1 (see Lemma 4.1 in [17]).

### Lemma 4.1

*Let* $(\mu_{0},u_{0})$ *be an approximation for* $(\mu_{k},u_{k})$, *where* $\mu_{0}$ *is not an eigenvalue of* $T_{h}$, *and* $u_{0}\in S^{h}$ *with* $\Vert u_{0} \Vert _{h}=1$. *Suppose that*

(C1): $\operatorname{dist} (u_{0},M_{h}(\mu_{k}) )\leq \frac{1}{2}$;
(C2): $\vert \mu_{0}-\mu_{k} \vert \leq\frac{\rho}{4}, \vert \mu_{j,h}-\mu_{j} \vert \leq\frac{\rho}{4}$ *for* $j=k-1,k,k+q$ ($j\neq0$), *where* $\rho=\min_{\mu_{j}\neq\mu_{k}}|\mu_{j}-\mu_{k}|$ *is the separation constant of the eigenvalue* $\mu_{k}$;
(C3): $u'\in S^{h},u_{k}^{h}\in S^{h}$ *satisfy*
  $$(\mu_{0}-T_{h})u'=u_{0},\qquad u_{k}^{h}= \frac{u'}{ \Vert u \Vert _{h}}, $$
  *then the following inequality holds*:
  4.1 $$ \operatorname{dist} \bigl(u_{k}^{h},M_{h}( \mu_{k}) \bigr) \leq\frac{4}{\rho}\max_{k\leq j\leq k+q-1} \vert \mu_{0}-\mu_{j,h} \vert \operatorname{dist} \bigl(u_{0},M_{h}(\mu_{k}) \bigr). $$

Next, we will use the proof method in [17] to analyze the error of Schemes 1–2.

Let $\delta_{0}$ be a positive constant satisfying the following inequalities:

4.2 $$\begin{aligned} &\max \{1,C_{5} \}\delta_{0}\leq\min \biggl\{ \frac{1}{2},\frac{1}{4C_{3}\sqrt{\lambda_{k}}} \biggr\} ; \end{aligned}$$

4.3 $$\begin{aligned} &4C_{3}C_{7}\delta_{0}^{2}+4C_{3}^{2} \lambda_{k}\delta_{0}+2\lambda_{k} \delta_{0}+C_{6}\delta_{0}\leq \frac{1}{2}; \end{aligned}$$

4.4 $$\begin{aligned} &\frac{\delta_{0}}{(\lambda_{k}-\delta_{0})\lambda_{k}}\leq\frac{\rho}{4},\quad \delta_{0}\leq \frac{\lambda_{k}}{2} ; \end{aligned}$$

4.5 $$\begin{aligned} &\frac{C_{5}^{2}C_{7}\delta_{0}^{2}}{\lambda_{j}(\lambda_{j}-C_{5}^{2}C_{7}\delta_{0}^{2})}\leq\frac{\rho}{4},\quad j=k-1,k,\ldots,k+q,j \neq0. \end{aligned}$$

### Condition 4.1

There exists $\bar{u}\in M(\lambda_{k})$ such that

$$\bigl\Vert u^{h_{l}}-\bar{u} \bigr\Vert _{h}\leq \delta_{0},\qquad \vert \lambda_{0}-\lambda_{k} \vert \leq \delta_{0},\qquad \delta_{h_{l}}( \lambda_{j})\leq\delta_{0}\quad (j=k-1,k,k+1,j\neq0), $$

where $\lambda_{0}$ is an approximate eigenvalue of $\lambda_{k}$, $u^{h_{l}}$ is an approximate eigenfunction obtained by Scheme 1 or Scheme 2, and *ρ* is the separation constant of the eigenvalue $\mu_{k}=\frac{1}{\lambda_{k}}$.

Condition 4.1 plays a key role in proving Theorem 4.1, by which we can prove Theorems 4.2–4.3. In the proof of Theorems 4.2–4.3, we can deduce that Condition 4.1 holds when the mesh size *H* is appropriately small. However, it is difficult to verify the condition whether the mesh size *H* is appropriately small or not. And it seems to be a necessary condition in many papers on the convergence and error estimates of the finite element method for eigenvalue problem. But numerical experiments in Sect. 6 present a satisfying practical performance for our algorithms, which shows that it is unnecessary for the mesh size *H* to be appropriately small, even though the theory is not complete.

The following Theorems 4.1–4.3 are the generalization of Theorems 4.2–4.4 in [17].

### Theorem 4.1

*Let* $(\lambda_{k}^{h_{l}},u_{k}^{h_{l}})$ *be an approximate eigenvalue obtained by Scheme* 1 *or Scheme *2. *Assume that Lemma* 2.1 *and Condition* 4.1 *hold with* $\lambda_{0}=\lambda_{k}^{h_{l-1}}$ *for Scheme* 1 *or* $\lambda_{0}=\lambda_{k}^{h_{i0}}$ *for Scheme* 2. *Then there exists* $u_{k}\in M(\lambda_{k})$ *such that*

4.6 $$ \bigl\Vert u_{k}^{h_{l}}-u_{k} \bigr\Vert _{h} \leq\frac{C_{0}}{2} \bigl\{ \vert \lambda_{0}-\lambda_{k} \vert \bigl( \bigl\vert \lambda_{k}^{h_{l-1}}-\lambda_{k} \bigr\vert + \bigl\Vert u_{k}^{h_{l-1}}-\bar{u} \bigr\Vert _{h} \bigr)+\delta_{h_{l}}(\lambda_{k}) \bigr\} . $$

### Proof

We use Lemma 4.1 to complete the proof. Select $\mu_{0}=\frac{1}{\lambda_{0}}$ and $u_{0}=\frac{\lambda_{k}^{h_{l-1}}T_{h_{l}}u_{k}^{h_{l-1}}}{ \Vert \lambda_{k}^{h_{l-1}}T_{h_{l}}u_{k}^{h_{l-1}} \Vert _{h}}$. Then, by (2.6) and (2.8), we have

$$\begin{aligned} & \bigl\Vert \lambda_{k}^{h_{l-1}}T_{h_{l}}u_{k}^{h_{l-1}}- \bar{u} \bigr\Vert _{h} \\ &\quad = \bigl\Vert \lambda_{k}^{h_{l-1}}T_{h_{l}}u_{k}^{h_{l-1}}- \lambda_{k}T_{h_{l}}u_{k}^{h_{l-1}}+ \lambda_{k}T_{h_{l}}u_{k}^{h_{l-1}}- \lambda_{k}T_{h_{l}}\bar{u}+\lambda_{k}T_{h_{l}} \bar{u}-\lambda_{k}T\bar{u} \bigr\Vert _{h} \\ &\quad \leq C_{3} \bigl\vert \lambda_{k}^{h_{l-1}}- \lambda_{k} \bigr\vert +C_{3}\lambda_{k} \bigl\Vert u_{k}^{h_{l-1}}-\bar{u} \bigr\Vert _{0}+ \lambda_{k} \bigl\Vert (T_{h_{l}}-T)|_{M(\lambda_{k})} \bigr\Vert _{h} \Vert \bar{u} \Vert _{h} \\ &\quad \leq C_{3} \bigl\vert \lambda_{k}^{h_{l-1}}- \lambda_{k} \bigr\vert +C_{3}^{2} \lambda_{k} \bigl\Vert u_{k}^{h_{l-1}}-\bar{u} \bigr\Vert _{h}+\lambda_{k}\delta_{h_{l}}( \lambda_{k}) \Vert \bar{u} \Vert _{h}. \end{aligned}$$

Noting that $\Vert \bar{u} \Vert _{h}\geq \Vert u_{k}^{h_{l-1}} \Vert _{h}- \Vert \bar{u}-u_{k}^{h_{l-1}} \Vert _{h}\geq1-\delta_{0}\geq\frac{1}{2}$, thus, by Lemma 3.1 in [26], we have

4.7 $$\begin{aligned} \biggl\Vert u_{0}-\frac{\bar{u}}{ \Vert \bar{u} \Vert _{h}} \biggr\Vert _{h}&\leq\frac{2}{ \Vert \bar{u} \Vert _{h}} \bigl\Vert \lambda_{k}^{h_{l-1}}T_{h_{l}}u_{k}^{h_{l-1}}- \bar{u} \bigr\Vert _{h} \\ &\leq 4C_{3} \bigl\vert \lambda_{k}^{h_{l-1}}- \lambda_{k} \bigr\vert +4C_{3}^{2} \lambda_{k} \bigl\Vert u_{k}^{h_{l-1}}-\bar{u} \bigr\Vert _{h}+2\lambda_{k}\delta_{h_{l}}( \lambda_{k}). \end{aligned}$$

Using the triangle inequality, (4.7), (2.11), Condition 4.1 and (4.3), we get

4.8 $$\begin{aligned} &\operatorname{dist} \bigl(u_{0},M_{h_{l}}( \lambda_{k}) \bigr) \\ &\quad \leq \biggl\Vert u_{0}-\frac{\bar{u}}{ \Vert \bar{u} \Vert _{h}} \biggr\Vert _{h}+\operatorname{dist} \biggl(\frac{\bar{u}}{ \Vert \bar{u} \Vert _{h}},M_{h_{l}}( \lambda_{k}) \biggr) \\ &\quad \leq 4C_{3} \bigl\vert \lambda_{k}^{h_{l-1}}- \lambda_{k} \bigr\vert +4C_{3}^{2} \lambda_{k} \bigl\Vert u_{k}^{h_{l-1}}-\bar{u} \bigr\Vert _{h}+2\lambda_{k}\delta_{h_{l}}( \lambda_{k})+C_{6}\delta_{h_{l}}( \lambda_{k}) \\ &\quad \leq 4C_{3}C_{7}\delta_{0}^{2}+4C_{3}^{2} \lambda_{k}\delta_{0}+2\lambda_{k} \delta_{0}+C_{6}\delta_{0}\leq \frac{1}{2}. \end{aligned}$$

From Condition 4.1, (4.4), we have

$$\vert \mu_{0}-\mu_{k} \vert =\frac{ \vert \lambda_{0}-\lambda_{k} \vert }{\lambda_{0}\lambda_{k}}\leq \frac{\delta_{0}}{(\lambda_{k}-\delta_{0})\lambda_{k}}\leq\frac{\rho}{4}. $$

From (2.13), we deduce

$$\begin{aligned} \vert \mu_{j}-\mu_{j,h_{l}} \vert =& \biggl\vert \frac{\lambda_{j}-\lambda_{j,h_{l}}}{\lambda_{j}\lambda_{j,h_{l}}} \biggr\vert \leq \frac{C_{5}^{2}C_{7}\delta_{h}(\lambda)^{2}}{\lambda_{j}(\lambda_{j}-C_{5}^{2}C_{7}\delta_{h}(\lambda)^{2})} \leq \frac{C_{5}^{2}C_{7}\delta_{0}^{2}}{\lambda_{j}(\lambda_{j}-C_{5}^{2}C_{7}\delta_{0}^{2})}\leq\frac{\rho}{4}. \end{aligned}$$

Hence, the conditions in Lemma 4.1 are verified.

By (2.5) we see that Step 3 in Scheme 1 or Step 6 in Scheme 2 is equivalent to the following:

$$a_{h} \bigl(u',v \bigr)-\lambda_{0}a_{h} \bigl(T_{h_{l}}u',v \bigr)=a_{h} \bigl(T_{h_{l}}u_{k}^{h_{l-1}},v \bigr),\quad \forall v\in S^{h_{l}}, $$

$u_{k}^{h_{l}}=\frac{u'}{ \Vert u' \Vert _{h}}$, i.e.,

$$\bigl(\lambda_{0}^{-1}-T_{h_{l}} \bigr)u'=\lambda_{0}^{-1}T_{h_{l}}u_{k}^{h_{l-1}}, \quad u_{k}^{h_{l}}=\frac{u'}{ \Vert u' \Vert _{h}}. $$

Then Step 3 in Scheme 1 or Step 6 in Scheme 2 is equivalent to

$$\bigl(\lambda_{0}^{-1}-T_{h_{l}} \bigr)u'=u_{0},\quad u_{k}^{h_{l}}= \frac{u'}{ \Vert u' \Vert _{h}}. $$

From (4.4), (2.13) and (4.5), we derive that

4.9 $$\begin{aligned} \vert \mu_{0}-\mu_{j,h_{l}} \vert =& \biggl\vert \frac{1}{\lambda_{0}}-\frac{1}{\lambda_{j,h_{l}}} \biggr\vert \leq \frac{4 \vert \lambda_{0}-\lambda_{j,h_{l}} \vert }{\lambda_{k}^{2}} \leq \frac{4}{\lambda_{k}^{2}} \vert \lambda_{0}-\lambda_{k} \vert +\frac{4}{\lambda_{k}^{2}} \vert \lambda_{k}-\lambda_{j,h_{l}} \vert \\ \leq& \frac{4}{\lambda_{k}^{2}}\delta_{0}+\frac{4C_{5}^{2}C_{7}}{\lambda_{k}^{2}} \delta_{0}^{2},\quad j=k,k+1,\ldots,k+q-1. \end{aligned}$$

Let the eigenvectors $\{u_{j,h_{l}}\}_{k}^{k+q-1}$ be an orthogonal basis of $M_{h_{l}}(\lambda_{k})$ with respect to $a_{h}(\cdot,\cdot)$. Denote

$$u^{*}=\sum_{j=k}^{k+q-1}a_{h} \bigl(u_{k}^{h_{l}},u_{j,h_{l}} \bigr)u_{j,h_{l}}, $$

then

$$\bigl\Vert u_{k}^{h_{l}}-u^{*} \bigr\Vert _{h}= \operatorname{dist} \bigl(u_{k}^{h_{l}},M_{h_{l}}( \lambda_{k}) \bigr). $$

Hence, substituting (4.8) and (4.9) into (4.1), we obtain

4.10 $$\begin{aligned} \bigl\Vert u_{k}^{h_{l}}-u^{*} \bigr\Vert _{h} =&\operatorname{dist} \bigl(u_{k}^{h_{l}},M_{h_{l}}( \lambda_{k}) \bigr) \\ \leq &\frac{4}{\rho} \biggl(\frac{4}{\lambda_{k}^{2}} \vert \lambda_{0}-\lambda_{k} \vert +\frac{4C_{5}^{2}C_{7}}{\lambda_{k}^{2}} \delta_{h}^{2}(\lambda) \biggr) \\ &{}\times \bigl(4C_{3} \bigl\vert \lambda_{k}^{h_{l-1}}- \lambda_{k} \bigr\vert +4C_{3}^{2} \lambda_{k} \bigl\Vert u_{k}^{h_{l-1}}-\bar{u} \bigr\Vert _{h} \\ &{}+2\lambda_{k}\delta_{h_{l}}( \lambda_{k})+C_{6}\delta_{h_{l}}( \lambda_{k}) \bigr). \end{aligned}$$

By Lemma 2.1, there exist eigenvectors $\{u_{j}^{0} \}_{k}^{k+q-1}$ making $u_{j,h_{l}}$ and $u_{j}^{0}$ satisfy (2.10). Let

$$u_{k}=\sum_{j=k}^{k+q-1}a_{h} \bigl(u_{k}^{h_{l}},u_{j,h_{l}} \bigr)u_{j}^{0}, $$

then $u_{k}\in M(\lambda_{k})$.

Using (2.10), we deduce that

$$\begin{aligned} \bigl\Vert u_{k}-u^{*} \bigr\Vert _{h} =& \Biggl\Vert \sum_{j=k}^{k+q-1}a_{h} \bigl(u_{k}^{h_{l}},u_{j,h_{l}} \bigr) \bigl(u_{j}^{0}-u_{j,h_{l}} \bigr) \Biggr\Vert _{h} \\ \leq & \Biggl(\sum_{j=k}^{k+q-1} \bigl\Vert u_{j}^{0}-u_{j,h_{l}} \bigr\Vert _{h}^{2} \Biggr)^{\frac{1}{2}}\leq \bigl(C_{5}^{2} \delta_{h_{l}}(\lambda_{j})^{2} \bigr)^{\frac{1}{2}} \leq q^{\frac{1}{2}}C_{5}\delta_{h_{l}}(\lambda_{j}). \end{aligned}$$

Noting that the constants $C_{3},C_{5},C_{6},C_{7}$ and *ρ* are independent of mesh parameters and iterative times *l*, and $\Vert u_{k}^{h_{l-1}}-\bar{u} \Vert _{h}\leq\delta_{0}, \vert \lambda_{0}-\lambda_{k} \vert \leq\delta_{0}$ and $\delta_{h_{l}}(\lambda_{k})\leq\delta_{0}$, by (4.10) and (4.2), we know that there exists a positive constant $C_{0}$ that is independent of mesh parameters and *l* such that (4.6) holds. And we can have $C_{0}\geq C_{5}$. □

We need the following two conditions (see Conditions 4.2 and 4.3 in [17]).

### Condition 4.2

There exists $t_{i}\in(1,2]$ ($i=1,2,\ldots $) such that $\delta_{h_{i}}(\lambda_{k})=\delta_{h_{i-1}}^{t_{i}}(\lambda_{k})$ and $\delta_{h_{i}}(\lambda_{k})\rightarrow 0$ ($i\rightarrow\infty$).

Condition 4.2 is easily satisfied; for example, for smooth eigenfunction, by using the uniform mesh, choose $h_{0}=\frac{\sqrt{2}}{8}$, $h_{1}=\frac{\sqrt{2}}{32}$, $h_{2}=\frac{\sqrt{2}}{64}$, and $h_{3}=\frac{\sqrt{2}}{128}$; then we have $h_{i}=h^{t_{i}}_{i-1}$, i.e., $\delta_{h_{i}}(\lambda_{k})=\delta^{t_{i}}_{h_{i}-1}(\lambda_{k})$, where $t_{1}\approx1.80,t_{2}\approx1.22,t_{3}\approx1.18$. For a nonsmooth eigenfunction, the condition could be met when the local refinement is done near the singular point.

### Condition 4.3

For any given number $\beta_{0}\in(0,1)$, there exists $0<\beta_{0}\leq\beta_{i}<1$ ($i=1,2,\ldots$) such that $\delta_{h_{i}}(\lambda_{k})=\beta_{i}\delta_{h_{i-1}}(\lambda_{k}),\delta_{h_{i}}(\lambda_{k})\rightarrow 0$ ($i\rightarrow \infty$).

### Theorem 4.2

*Let* $(\lambda_{k}^{h_{l}},u_{k}^{h_{l}})$ *be an approximate eigenpair obtained by Scheme* 1. *Suppose that Condition* 4.2 *holds*, *then there exist* $u_{k}\in M(\lambda_{k})$ *and* $H_{0}>0$ *such that if* $H< H_{0}$, *Lemma* 2.1 *and the following estimates hold*:

4.11 $$\begin{aligned} & \bigl\Vert u_{k}^{h_{l}}-u_{k} \bigr\Vert _{h}\leq C_{0}\delta_{h_{l}}(\lambda_{k}), \end{aligned}$$

4.12 $$\begin{aligned} & \bigl\vert \lambda_{k}^{h_{l}}-\lambda_{k} \bigr\vert \leq C_{0}^{1+r}C_{7} \delta_{h_{l}}^{1+r}(\lambda_{k}). \end{aligned}$$

### Proof

The proof is completed by using induction and Theorem 4.1 with $\lambda_{0}=\lambda_{k}^{h_{l-1}}$. Note that $\delta_{H}(\lambda_{k})\rightarrow0$, then there is a proper small $H_{0}>0$ such that if $H\leq H_{0}$, Lemma 2.1 and the following inequalities hold:

4.13 $$\begin{aligned} &C_{0}\delta_{H}(\lambda_{k})\leq \delta_{0},\qquad C_{0}^{1+r}C_{7} \delta_{H}^{1+r}(\lambda_{k})\leq \delta_{0}, \end{aligned}$$

4.14 $$\begin{aligned} &C_{0}^{2+2r}C_{7}^{2} \delta_{H}^{2r}(\lambda_{k})+C_{0}^{2+r}C_{7} \delta_{H}^{r}(\lambda_{k})\leq 1. \end{aligned}$$

When $l=1$, we have $(\lambda_{k}^{h_{l-1}},u_{k}^{h_{l-1}})=(\lambda_{k,H},u_{k,H})$; from Lemma 2.1 and (2.12), we know that there exists $\bar{u}\in M(\lambda_{k})$ such that

$$\begin{aligned} & \Vert u_{k,H}-\bar{u} \Vert _{H}\leq C_{5} \delta_{H}(\lambda_{k})\leq \delta_{0}, \\ & \vert \lambda_{k,H}-\lambda_{k} \vert \leq C_{5}^{1+r}C_{7}\delta_{H}^{1+r}( \lambda_{k})\leq\delta_{0}, \end{aligned}$$

and $\delta_{h_{1}}(\lambda{_{j}})\leq\delta_{0}$ ($j=k-1,k,k+q,j\neq 0$), i.e. Condition 4.1 holds. Thus, by Theorem 4.1 and $2-t_{1}\geq0$ and $C_{5}\leq C_{0}$ we get

$$\begin{aligned} \bigl\Vert u_{k}^{h_{1}}-u_{k} \bigr\Vert _{h} \leq& \frac{C_{0}}{2} \bigl\{ C_{5}^{2+2r}C_{7}^{2} \delta_{H}^{2+2r}(\lambda_{k})+C_{5}^{2+r}C_{7} \delta_{H}^{2+r}(\lambda_{k})+\delta_{h_{1}}( \lambda_{k}) \bigr\} \\ \leq& \frac{C_{0}}{2} \bigl\{ C_{0}^{2+2r}C_{7}^{2} \delta_{H}^{2+2r-t_{1}}(\lambda_{k})+C_{0}^{2+r}C_{7} \delta_{H}^{2+r-t_{1}}(\lambda_{k})+1 \bigr\} \delta_{h_{1}}(\lambda_{k}) \\ \leq& \frac{C_{0}}{2} \bigl\{ C_{0}^{2+2r}C_{7}^{2} \delta_{H}^{2r}(\lambda_{k})+C_{0}^{2+r}C_{7} \delta_{H}^{r}(\lambda_{k})+1 \bigr\} \delta_{h_{1}}(\lambda_{k}) \\ \leq& C_{0}\delta_{h_{1}}(\lambda_{k}). \end{aligned}$$

Combining (2.12) and the above inequality yields

$$\bigl\vert \lambda_{k}^{h_{1}}-\lambda_{k} \bigr\vert \leq C_{7} \bigl\Vert u_{k}^{h_{1}}-u_{k} \bigr\Vert _{h}^{1+r}\leq C_{0}^{1+r}C_{7} \delta_{h_{1}}^{1+r}(\lambda_{k}). $$

Suppose that Theorem 4.2 is valid for $l-1$, i.e. there exists $\bar{u}\in M(\lambda_{k})$ such that

$$\begin{aligned} & \bigl\Vert u_{k}^{h_{l-1}}-\bar{u} \bigr\Vert _{h} \leq C_{0}\delta_{h_{l-1}}(\lambda_{k}), \\ & \bigl\vert \lambda_{k}^{h_{l-1}}-\lambda_{k} \bigr\vert \leq C_{0}^{1+r}C_{7} \delta_{h_{l-1}}^{1+r}(\lambda_{k}), \end{aligned}$$

then, owing to (4.13)–(4.14), we have $\Vert u_{k}^{h_{l-1}}-\bar{u} \Vert _{h} \leq\delta_{0}$ and $\vert \lambda_{k}^{h_{l-1}}-\lambda_{k} \vert \leq\delta_{0}$ ($j=k-1,k,k+q, j\neq0$), i.e. the conditions of Theorem 4.1 hold. Therefore, for *l*, by (4.6) and (4.14) we deduce

$$\begin{aligned} \bigl\Vert u_{k}^{h_{l}}-u_{k} \bigr\Vert _{h} \leq& \frac{C_{0}}{2} \bigl\{ C_{0}^{2+2r}C_{7}^{2} \delta_{h_{l-1}}^{2+2r}(\lambda_{k})+C_{0}^{2+r}C_{7} \delta_{h_{l-1}}^{2+r}(\lambda_{k})+ \delta_{h_{l}}(\lambda_{k}) \bigr\} \\ \leq& \frac{C_{0}}{2} \bigl\{ C_{0}^{2+2r}C_{7}^{2} \delta_{h_{l-1}}^{2+2r-t_{l}}(\lambda_{k})+C_{0}^{2+r}C_{7} \delta_{h_{l-1}}^{2+r-t_{l}}(\lambda_{k})+1 \bigr\} \delta_{h_{l}}(\lambda_{k}) \\ \leq& \frac{C_{0}}{2} \bigl\{ C_{0}^{2+2r}C_{7}^{2} \delta_{H}^{2+2r-t_{l}}(\lambda_{k})+C_{0}^{2+r}C_{7} \delta_{H}^{2+r-t_{l}}(\lambda_{k})+1 \bigr\} \delta_{h_{l}}(\lambda_{k}) \\ \leq& \frac{C_{0}}{2} \bigl\{ C_{0}^{2+2r}C_{7}^{2} \delta_{H}^{2r}(\lambda_{k})+C_{0}^{2+r}C_{7} \delta_{H}^{r}(\lambda_{k})+1 \bigr\} \delta_{h_{l}}(\lambda_{k}) \\ \leq& C_{0}\delta_{h_{l}}(\lambda_{k}). \end{aligned}$$

By (2.12) and the above inequality we deduce

$$\bigl\vert \lambda_{k}^{h_{l}}-\lambda_{k} \bigr\vert \leq C_{7} \bigl\Vert u_{k}^{h_{l}}-u_{k} \bigr\Vert _{h}^{1+r}\leq C_{0}^{1+r}C_{7} \delta_{h_{l}}^{1+r}(\lambda_{k}), $$

i.e. (4.11)–(4.12) are valid. □

### Theorem 4.3

*Let* $(\lambda_{k}^{h_{l}},u_{k}^{h_{l}})$ *be an approximate eigenpair obtained by Scheme* 2. *Suppose that Condition* 4.2 *holds for* $i\leq i_{0}$ *and Condition* 4.3 *holds for* $i>i_{0}$. *Then there exist* $u_{k}\in M(\lambda_{k})$ *and* $H_{0}>0$ *such that if* $H\leq H_{0}$ *it holds that*

4.15 $$\begin{aligned} & \bigl\Vert u_{k}^{h_{l}}-u_{k} \bigr\Vert _{h}\leq C_{0}\delta_{h_{l}}(\lambda_{k}), \end{aligned}$$

4.16 $$\begin{aligned} & \bigl\vert \lambda_{k}^{h_{l}}-\lambda_{k} \bigr\vert \leq C_{0}^{1+r}C _{7} \delta_{h_{l}}^{1+r}(\lambda_{k}),\quad l>i_{0}. \end{aligned}$$

### Proof

The proof is completed by using induction and Theorem 4.1 with $\lambda_{0}=\lambda_{k}^{h_{i_{0}}}$. Note that $\delta_{H}(\lambda_{k})\rightarrow 0$ ($H\rightarrow0$), then there is a proper small $H_{0}>0$ such that if $H\leq H_{0}$, Lemma 2.1 and the following inequalities hold:

4.17 $$\begin{aligned} &C_{0}\delta_{H}(\lambda_{k})\leq \delta_{0},\qquad C_{0}^{1+r}C_{7} \delta_{H}^{1+r}(\lambda_{k})\leq \delta_{0}, \end{aligned}$$

4.18 $$\begin{aligned} &C_{0}^{2+2r}C_{7}^{2} \delta_{h_{l_{0}+1}}^{1+r}(\lambda_{k})\delta_{h_{l-1}}^{r}( \lambda_{k})\frac{1}{\beta_{0}} +C_{0}^{2+r}C_{7} \delta_{h_{l_{0}+1}}^{1+r}(\lambda_{k})\frac{1}{\beta_{0}} \leq 1. \end{aligned}$$

When $l=i_{0}+1$, by Theorem 4.2 we know that there exists $u_{k}\in M(\lambda_{k})$ such that

$$\begin{aligned} & \bigl\Vert u_{k}^{h_{i_{0}+1}}-u_{k} \bigr\Vert _{h}\leq C_{0}\delta_{h_{i_{0}+1}}(\lambda_{k}), \\ & \bigl\vert \lambda_{k}^{h_{i_{0}+1}}-\lambda_{k} \bigr\vert \leq C_{0}^{1+r}C_{7} \delta_{h_{i_{0}+1}}^{1+r}(\lambda_{k}). \end{aligned}$$

Suppose that Theorem 4.3 holds for $l-1$, i.e. there exists $\bar{u}\in M(\lambda_{k})$ such that

$$\begin{aligned} & \bigl\Vert u_{k}^{h_{l-1}}-\bar{u} \bigr\Vert _{h}\leq C_{0}\delta_{h_{l-1}}(\lambda_{k}), \\ & \bigl\vert \lambda_{k}^{h_{l-1}}-\lambda_{k} \bigr\vert \leq C_{0}^{1+r}C_{7} \delta_{h_{l-1}}^{1+r}(\lambda_{k}). \end{aligned}$$

Then we infer from (4.17) that the conditions of Theorem 4.1 hold; therefore, for *l*, we can get

$$\begin{aligned} & \bigl\Vert u_{k}^{h_{l}}-u_{k} \bigr\Vert _{h} \\ &\quad \leq \frac{C_{0}}{2} \bigl\{ C_{0}^{2+2r}C_{7}^{2} \delta_{h_{l_{0}+1}}^{1+r}(\lambda_{k}) \delta_{h_{l-1}}^{1+r}( \lambda_{k}) +C_{0}^{2+r}C_{7} \delta_{h_{l_{0}+1}}^{1+r}(\lambda_{k}) \delta_{h_{l-1}}( \lambda_{k})+\delta_{h_{l}}( \lambda_{k}) \bigr\} \\ &\quad \leq \frac{C_{0}}{2} \biggl\{ C_{0}^{2+2r}C_{7}^{2} \delta_{h_{l_{0}+1}}^{1+r}(\lambda_{k}) \delta_{h_{l-1}}^{r}( \lambda_{k})\frac{1}{\beta_{0}} +C_{0}^{2+r}C_{7} \delta_{h_{l_{0}+1}}^{1+r}( \lambda_{k})\frac{1}{\beta_{0}}+1 \biggr\} \delta_{h_{l}}( \lambda_{k}), \end{aligned}$$

which together with (4.18), we get (4.15). Substituting (4.15) into the inequality (2.12), we get (4.16). □

### Remark

For some adaptive local refined grids used usually, (2.9) can be expressed as $\vert E_{h}(u,v) \vert \leq C_{4}h \Vert v \Vert _{h}$, $\forall v\in S^{h}+H_{0}^{2}(\Omega)$, therefore *r* in the theorems of this paper can take 1.

## Adaptive algorithms

In this section, referring to [10, 17, 27], we present six algorithms. We denote Algorithm 1 in [10] as Algorithm 1 in this paper, and Algorithms 2–3 are established based on Schemes 1–2, respectively. Then we combine Algorithms 1–3 with a uniformly refined algorithm to get Algorithms 1M–3M, respectively. And the a posterior error estimator in the following algorithms comes from [4], that is

5.1 $$\begin{aligned} &\begin{aligned} \eta_{h}(f,w_{h}, \kappa)^{2}={}&h_{\kappa}^{4} \Vert f \Vert _{0,\kappa}^{2} \\ &{}+\sum_{F\in \varepsilon_{h}\cap\partial\kappa}h_{F} \biggl\Vert \frac{1}{2} \bigl[ \bigl({\nabla(\nabla w_{h})+\nabla(\nabla w_{h})^{T}} \bigr)\tau_{F} \bigr] \biggr\Vert _{0,F}^{2} \quad \mbox{in }\mathbb{R}^{2}, \end{aligned} \\ &\begin{aligned} \eta_{h}(f,w_{h},\kappa)^{2}={}&h_{\kappa}^{4} \Vert f \Vert _{0,\kappa}^{2} \\ &{}+\sum_{F\in \varepsilon_{h}\cap\partial\kappa}h_{F} \biggl\Vert \frac{1}{2} \bigl[ \bigl({\nabla(\nabla w_{h})+\nabla(\nabla w_{h})^{T}} \bigr)\times\gamma_{F} \bigr] \biggr\Vert _{0,F}^{2}\quad \mbox{in }\mathbb{R}^{3}, \end{aligned} \\ &\eta_{h}(f,w_{h},\pi_{h})^{2}=\sum _{\kappa\in\pi_{h}}\eta_{h}(f,w_{h}, \kappa)^{2}, \end{aligned}$$

where $w_{h}$ is the finite element approximate solution of (2.4), $\tau_{F}$ is the tangential vector and $\gamma_{F}$ the unit outward normal on $F\in\varepsilon_{h}$.

In the following algorithms, we have to provide an initial shape regular triangulation $\pi_{h_{0}}$ and a parameter $\theta \in (0,1)$. Also, from [10, 11] we know that replacing $w_{h}$ with $u_{h}$ and replacing *f* with $\lambda_{h}u_{h}$ in (5.1), we can obtain the error estimator of Algorithms 1 and 1M. By Lemma 4.1 we can deduce that replacing $w_{h}$ with $u^{h}$ and replacing *f* with $\lambda^{h}u^{h}$ in (5.1), we can obtain the error estimator of Algorithms 2–3 and Algorithms 2M–3M.

### Algorithm 1

Choose the parameter $0<\theta<1$.

*Step 1.* Pick any initial mesh $\pi_{h_{0}}$.

*Step 2.* Solve (2.3) on $\pi_{h_{0}}$ for discrete solution $(\lambda_{h_{0}},u_{h_{0}})$.

*Step 3.* $l\Leftarrow0$.

*Step 4.* Compute the local indicators $\eta_{h_{l}}(\lambda_{h_{l}}u_{h_{l}},u_{h_{l}},\kappa)$.

*Step 5.* Construct $\hat{\pi}_{h_{l}}\in \pi_{h_{l}}$ by *Marking strategy* E and *θ*.

*Step 6.* Refine $\pi_{h_{l}}$ to get a new mesh $\pi_{h_{l+1}}$ by procedure *Refine*.

*Step 7.* Solve (2.3) on $\pi_{h_{l+1}}$ for discrete solution $(\lambda_{h_{l+1}},u_{h_{l+1}})$.

*Step 8.* $l\Leftarrow l+1$ and go to Step 4.

### Algorithm 2

Choose the parameter $0<\theta<1$.

*Step 1.* Pick any initial mesh $\pi_{h_{0}}$.

*Step 2.* Solve (2.3) on $\pi_{h_{0}}$ for discrete solution $(\lambda_{h_{0}},u_{h_{0}})$.

*Step 3.* $l\Leftarrow0, \lambda_{0}\Leftarrow\lambda_{h_{0}}, u^{h_{0}}\Leftarrow u_{h_{0}}$.

*Step 4.* Compute the local indicators $\eta_{h_{l}}(\lambda^{h_{l}}u^{h_{l}},u^{h_{l}},\kappa)$.

*Step 5.* Construct $\hat{\pi}_{h_{l}}\in \pi_{h_{l}}$ by *Marking strategy* E1 and *θ*.

*Step 6.* Refine $\pi_{h_{l}}$ to get a new mesh $\pi_{h_{l+1}}$ by procedure *Refine*.

*Step 7.* Find $u'\in V_{h_{l+1}}$ such that

5.2 $$ a_{h} \bigl(u',v \bigr)- \lambda_{0}b \bigl(u',v \bigr)=b \bigl(u^{h_{l}},v \bigr); $$

denote $u^{h_{l+1}}=\frac{u'}{ \Vert u' \Vert _{h}}$ and compute the Rayleigh quotient:

$$\lambda^{h_{l+1}}=\frac{a_{h} (u^{h_{l+1}},u^{h_{l+1}} )}{b (u^{h_{l+1}},u^{h_{l+1}} )}. $$

*Step 8.* $\lambda_{0}\Leftarrow\lambda^{h_{l+1}},l\Leftarrow l+1$ and go to Step 4.

### Algorithm 3

Choose the parameter $0<\theta<1$ and an integer $i_{0}$.

*Step 1*∼*Step 7.* The same as Steps 1–7 of Algorithm 2.

*Step 8.* If $l< i_{0},\lambda_{0}\Leftarrow\lambda^{h_{l+1}},l\Leftarrow l+1$ and go to Step 4; else $l\Leftarrow l+1$, and go to Step 4.

A family of good adaptive meshes should satisfy $h=O(h_{\min}^{\alpha})$. Hence, we give a bound $C_{r}$ of $\frac{h}{h_{\min}^{\alpha}}$. When the rate $\frac{h}{h_{\min}^{\alpha}}\geq C_{r}$ in the process of Algorithms 1M–3M is running, we refine the mesh uniformly for one time. And thus the following three algorithms are derived.

### Algorithm 1M

Choose the parameter $0<\theta<1$, *α*, and a bound $C_{r}$ of $\frac{h_{l}}{h_{l_{\min}}^{\alpha}}$.

*Step 1*∼*Step 7.* The same as Steps 1–7 of Algorithm 1.

*Step 8.* $l\Leftarrow l+1$.

*Step 9.* If $\frac{h_{l}}{h_{l_{\min}}^{\alpha}}\geq C_{r}$, then uniformly refine the mesh $\pi_{h_{l}}$ to get a new mesh $\pi_{h_{l+1}}$ and go to Step 7, else go to Step 4.

### Algorithm 2M

Choose the parameter $0<\theta<1$, *α*, and a bound $C_{r}$ of $\frac{h_{l}}{h_{l_{\min}}^{\alpha}}$.

*Step 1*∼*Step 7.* The same as Steps 1–7 of Algorithm 2.

*Step 8.* $\lambda_{0}\Leftarrow\lambda^{h_{l+1}},l\Leftarrow l+1$.

*Step 9.* If $\frac{h_{l}}{h_{l_{\min}}^{\alpha}}\geq C_{r}$, then uniformly refine the mesh $\pi_{h_{l}}$ to get a new mesh $\pi_{h_{l+1}}$ and go to Step 7, else go to Step 4.

### Algorithm 3M

Choose the parameter $0<\theta<1$, an integer $i_{0}$, *α*, and a bound $C_{r}$ of $\frac{h_{l}}{h_{l_{\min}}^{\alpha}}$.

*Step 1*∼*Step 7.* The same as Steps 1–7 of Algorithm 2.

*Step 8.* If $l< i_{0},\lambda_{0}\Leftarrow\lambda^{h_{l+1}},l\Leftarrow l+1$; else $l\Leftarrow l+1$.

*Step 9.* If $\frac{h_{l}}{h_{l_{\min}}^{\alpha}}\geq C_{r}$, then uniformly refine the mesh $\pi_{h_{l}}$ to get a new mesh $\pi_{h_{l+1}}$ and go to Step 7, else go to Step 4.

### Marking strategy E

Given parameter $0<\theta<1$:

*Step 1.* Construct a minimal subset $\widehat{\pi}_{h_{l}}$ of $\pi_{h_{l}}$ by selecting some elements in $\pi_{h_{l}}$ such that

$$\begin{aligned} \sum_{\kappa\in \widehat{\pi}_{h_{l}}}{\eta}_{h_{l}}^{2}( \lambda_{h_{l}}u_{h_{l}}, u_{h_{l}},\kappa) \geq \theta{ \eta}_{h_{l}}^{2}(\lambda_{h_{l}}u_{h_{l}},u_{h_{l}}, \Omega). \end{aligned}$$

*Step 2.* Mark all the elements in $\widehat{\pi}_{h_{l}}$.

### Marking strategy E1

To get Marking strategy E1 we only replace $\lambda_{h_{l}}$ and $u_{h_{l}}$ in Marking strategy E with $\lambda^{h_{l}}$ and $u^{h_{l}}$, respectively.

Algorithms 1M–3M including steps with uniform refinement seem to be opposite to the adaptive concept. Indeed, the combination of adaptive algorithms and uniform refinement meets the certain mesh-grading properties, thus improving the efficiency of Algorithms 1–3 (see Tables 1–3 in Sect. 6).

**Table 1:** The smallest eigenvalue solved by Algorithm 1 and Algorithm 1M

*l*	$N_{\mathrm{dof}}$	$h_{l}$	$\frac{h_{l}}{h_{l_{\min}}^{\alpha}}$	$\lambda_{1,h_{l}}$	CPU(*s*)	$N_{\mathrm{dof}}$	$h_{l}$	$\frac{h_{l}}{h_{l_{\min}}^{\alpha}}$	$\lambda_{1,h_{l}}^{M}$	CPU(*s*)
1	2945	0.044	0.210	6333.637	0.275	2945	0.044	0.210	6333.637	0.086
2	2957	0.044	0.250	6368.756	0.368	2957	0.044	0.250	6368.756	0.162
3	3035	0.044	0.297	6426.312	0.452	3035	0.044	0.297	6426.312	0.239
4	3135	0.044	0.354	6459.396	0.537	3135	0.044	0.354	6459.396	0.320
5	3345	0.044	0.420	6506.181	0.629	3345	0.044	0.420	6506.181	0.405
6	3609	0.044	0.500	6540.464	0.726	3609	0.044	0.500	6540.464	0.499
7	3979	0.044	0.595	6574.027	0.834	3979	0.044	0.595	6574.027	0.605
8	4459	0.044	0.707	6588.671	0.957	4459	0.044	0.707	6588.671	0.723
9	5097	0.044	0.841	6606.244	1.10	5097	0.044	0.841	6606.244	0.860
10	5787	0.044	1.00	6615.776	1.27	5787	0.044	1.00	6615.776	1.02
11	6665	0.044	1.19	6631.992	1.48	6665	0.044	1.19	6631.992	1.21
12	7791	0.044	1.41	6642.939	1.71	31,697	0.022	0.841	6688.240	2.12
13	9110	0.044	1.68	6656.854	1.97	34,833	0.022	1.00	6690.595	3.15
14	10,591	0.044	2.00	6662.468	2.27	39,191	0.022	1.19	6693.066	4.43
15	12,295	0.044	2.38	6665.980	2.65	173,919	0.011	0.841	6701.061	11.4
16	14,331	0.044	2.83	6671.824	3.10	189,989	0.011	1.00	6701.477	19.4
17	16,641	0.044	3.36	6676.967	3.62	211,977	0.011	1.19	6701.750	29.0
18	19,497	0.044	4.00	6680.844	4.21	948,969	0.006	0.841	6703.147	82.8
19	22,925	0.044	4.76	6684.502	4.92	1,025,149	0.006	1.00	6703.198	141
20	27,171	0.044	5.66	6686.546	5.77	1,131,177	0.006	1.19	6703.244	210
21	32,088	0.044	6.73	6689.797	6.76	5,114,697	0.003	0.841	6703.512	587
22	37,703	0.044	8.00	6692.349	7.95	–	–	–	–	–
23	44,289	0.044	9.51	6694.425	9.39	–	–	–	–	–
24	52,103	0.044	11.3	6695.960	11.1	–	–	–	–	–
25	60,857	0.044	13.5	6696.560	13.2	–	–	–	–	–
26	70,881	0.044	16.0	6697.400	15.7	–	–	–	–	–
27	83,091	0.031	13.5	6698.304	18.7	–	–	–	–	–
28	98,019	0.031	16.0	6699.274	22.7	–	–	–	–	–
29	116,273	0.031	19.0	6699.950	27.4	–	–	–	–	–
30	136,557	0.031	22.6	6700.589	33.1	–	–	–	–	–
31	160,465	0.022	16.0	6701.087	40.0	–	–	–	–	–
32	188,195	0.022	19.0	6701.469	48.2	–	–	–	–	–
33	221,401	0.022	22.6	6701.858	58.0	–	–	–	–	–
34	257,797	0.022	26.9	6702.060	69.7	–	–	–	–	–
35	301,063	0.022	32.0	6702.246	84.6	–	–	–	–	–
36	353,201	0.022	38.1	6702.411	102	–	–	–	–	–
37	416,609	0.022	45.3	6702.557	124	–	–	–	–	–
38	492,039	0.022	45.3	6702.767	151	–	–	–	–	–
39	577,233	0.022	53.8	6702.937	182	–	–	–	–	–
40	677,271	0.016	45.3	6703.035	220	–	–	–	–	–
41	793,765	0.016	53.8	6703.115	266	–	–	–	–	–
42	934,557	0.016	64.0	6703.190	321	–	–	–	–	–
43	1,084,193	0.016	64.0	6703.237	388	–	–	–	–	–
44	1,267,059	0.016	76.1	6703.272	465	–	–	–	–	–
45	1,487,051	0.016	90.5	6703.320	558	–	–	–	–	–
46	1,756,709	0.016	108	6703.362	672	–	–	–	–	–
47	2,065,245	0.011	90.5	6703.407	809	–	–	–	–	–
48	2,420,223	0.011	108	6703.446	973	–	–	–	–	–
49	2,834,373	0.011	128	6703.468	1171	–	–	–	–	–
50	3,319,763	0.011	128	6703.487	1415	–	–	–	–	–
51	3,894,763	0.011	152	6703.505	1706	–	–	–	–	–
52	4,522,239	0.011	181	6703.516	2060	–	–	–	–	–

**Table 2:** The smallest eigenvalue solved by Algorithm 2 and Algorithm 2M

*l*	$N_{\mathrm{dof}}$	$h_{l}$	$\frac{h_{l}}{h_{l_{\min}}^{\alpha}}$	$\lambda_{1,h_{l}}^{R}$	CPU(*s*)	$N_{\mathrm{dof}}$	$h_{l}$	$\frac{h_{l}}{h_{l_{\min}}^{\alpha}}$	$\lambda_{1,h_{l}}^{\mathrm{RM}}$	CPU(*s*)
1	2945	0.044	0.210	6333.637	0.133	2945	0.044	0.210	6333.637	0.090
2	2957	0.044	0.297	6373.503	0.228	2957	0.044	0.297	6373.503	0.140
3	3031	0.044	0.354	6538.971	0.280	3031	0.044	0.354	6538.971	0.192
4	3067	0.044	0.420	6443.737	0.371	3067	0.044	0.420	6443.737	0.250
5	3237	0.044	0.500	6489.761	0.426	3237	0.044	0.500	6489.761	0.305
6	3445	0.044	0.595	6523.466	0.487	3445	0.044	0.595	6523.466	0.365
7	3811	0.044	0.707	6566.620	0.560	3811	0.044	0.707	6566.620	0.431
8	4195	0.044	0.841	6595.431	0.636	4195	0.044	0.841	6595.431	0.504
9	4678	0.044	1.00	6597.955	0.720	4678	0.044	1.00	6597.955	0.588
10	5293	0.044	1.19	6607.441	0.814	5293	0.044	1.19	6607.441	0.709
11	6118	0.044	1.41	6623.248	0.924	25,297	0.022	0.841	6683.573	1.15
12	6997	0.044	1.68	6634.588	1.05	27,723	0.022	1.00	6686.324	1.63
13	8232	0.044	2.00	6648.804	1.20	30,933	0.022	1.19	6688.518	2.38
14	9527	0.044	2.38	6658.106	1.39	139,409	0.011	0.841	6700.069	5.87
15	11,102	0.044	2.83	6663.393	1.59	153,179	0.011	1.00	6700.762	9.71
16	12,928	0.044	3.36	6667.166	1.82	168,897	0.011	1.19	6701.161	15.4
17	15,139	0.044	4.00	6673.763	2.11	740,417	0.006	0.841	6702.983	39.1
18	17,619	0.044	4.76	6678.433	2.43	807,451	0.006	1.00	6703.097	65.4
19	20,763	0.044	5.66	6682.562	2.82	882,597	0.006	1.19	6703.149	103
20	24,365	0.044	6.73	6685.164	3.27	3,907,351	0.003	0.841	6703.486	236
21	28,967	0.044	8.00	6687.944	3.81	4,218,771	0.003	1.00	6703.504	382
22	34,068	0.044	9.51	6690.675	4.50	–	–	–	–	–
23	40,007	0.044	11.3	6692.914	5.39	–	–	–	–	–
24	47,117	0.044	13.5	6694.937	6.46	–	–	–	–	–
25	55,275	0.044	16.0	6696.294	7.64	–	–	–	–	–
26	64,407	0.044	19.0	6696.867	9.09	–	–	–	–	–
27	75,259	0.031	13.5	6697.823	10.8	–	–	–	–	–
28	88,353	0.031	16.0	6698.752	13.2	–	–	–	–	–
29	104,269	0.031	19.0	6699.457	16.1	–	–	–	–	–
30	123,285	0.031	22.6	6700.133	19.4	–	–	–	–	–
31	145,061	0.031	26.9	6700.774	23.3	–	–	–	–	–
32	170,397	0.022	22.6	6701.291	27.9	–	–	–	–	–
33	199,833	0.022	26.9	6701.638	33.5	–	–	–	–	–
34	235,261	0.022	26.9	6701.906	40.0	–	–	–	–	–
35	272,877	0.022	32.0	6702.117	48.6	–	–	–	–	–
36	319,549	0.022	38.1	6702.292	58.8	–	–	–	–	–
37	375,279	0.022	45.3	6702.462	71.1	–	–	–	–	–
38	444,149	0.022	53.8	6702.631	86.1	–	–	–	–	–
39	522,525	0.022	64.0	6702.819	104	–	–	–	–	–
40	613,375	0.022	76.1	6702.964	125	–	–	–	–	–
41	719,217	0.016	53.8	6703.062	149	–	–	–	–	–
42	844,333	0.016	64.0	6703.144	179	–	–	–	–	–
43	988,863	0.016	76.1	6703.208	214	–	–	–	–	–
44	1,150,057	0.016	90.5	6703.256	255	–	–	–	–	–
45	1,346,861	0.016	108	6703.292	304	–	–	–	–	–
46	1,584,041	0.016	128	6703.337	362	–	–	–	–	–
47	1,873,597	0.016	152	6703.378	433	–	–	–	–	–
48	2,196,067	0.011	108	6703.422	509	–	–	–	–	–
49	2,572,281	0.011	128	6703.454	598	–	–	–	–	–
50	3,010,543	0.011	152	6703.474	705	–	–	–	–	–
51	3,538,161	0.011	181	6703.497	831	–	–	–	–	–
52	4,120,833	0.011	215	6703.510	979	–	–	–	–	–

**Table 3:** The smallest eigenvalue solved by Algorithm 3 and Algorithm 3M

*l*	$N_{\mathrm{dof}}$	$h_{l}$	$\frac{h_{l}}{h_{l_{\min}}^{\alpha}}$	$\lambda_{1,h_{l}}^{F}$	CPU(*s*)	$N_{\mathrm{dof}}$	$h_{l}$	$\frac{h_{l}}{h_{l_{\min}}^{\alpha}}$	$\lambda_{1,h_{l}}^{FM}$	CPU(*s*)
1	2945	0.044	0.210	6333.637	0.086	2945	0.044	0.210	6333.637	0.086
2	2957	0.044	0.297	6373.503	0.137	2957	0.044	0.297	6373.503	0.137
3	3031	0.044	0.354	6538.971	0.187	3031	0.044	0.354	6538.971	0.187
4	3067	0.044	0.420	6443.737	0.244	3067	0.044	0.420	6443.737	0.245
5	3237	0.044	0.500	6489.761	0.299	3237	0.044	0.500	6489.761	0.300
6	3445	0.044	0.595	6523.466	0.358	3445	0.044	0.595	6523.466	0.360
7	3811	0.044	0.707	6566.620	0.423	3811	0.044	0.707	6566.620	0.426
8	4195	0.044	0.841	6595.431	0.496	4195	0.044	0.841	6595.431	0.498
9	4678	0.044	1.00	6597.955	0.577	4678	0.044	1.00	6597.955	0.581
10	5293	0.044	1.19	6607.441	0.670	5293	0.044	1.19	6607.441	0.696
11	6118	0.044	1.41	6623.248	0.779	25,297	0.022	0.841	6683.573	1.14
12	6997	0.044	1.68	6634.588	0.903	27,723	0.022	1.00	6686.324	1.62
13	8232	0.044	2.00	6648.804	1.05	30,933	0.022	1.19	6688.518	2.37
14	9527	0.044	2.38	6658.106	1.21	139,409	0.011	0.841	6700.069	5.85
15	11,102	0.044	2.83	6663.393	1.41	153,179	0.011	1.00	6701.996	9.71
16	12,928	0.044	3.36	6667.166	1.64	164,253	0.011	1.19	6701.274	15.2
17	15,139	0.044	4.00	6673.763	1.93	715,319	0.006	0.841	6702.994	38.2
18	17,619	0.044	4.76	6678.433	2.25	780,735	0.006	1.00	6703.096	63.8
19	20,763	0.044	5.66	6682.562	2.63	853,934	0.006	1.19	6703.205	100
20	24,365	0.044	6.73	6685.164	3.08	3,774,935	0.003	0.841	6703.503	231
21	28,967	0.044	8.00	6687.944	3.60	4,083,915	0.003	1.00	6703.538	372
22	34,068	0.044	9.51	6690.675	4.21	–	–	–	–	–
23	40,007	0.044	11.3	6692.914	4.97	–	–	–	–	–
24	47,117	0.044	13.5	6694.937	5.89	–	–	–	–	–
25	55,275	0.044	16.0	6696.294	6.98	–	–	–	–	–
26	64,407	0.044	19.0	6696.867	8.28	–	–	–	–	–
27	75,259	0.031	13.5	6697.823	9.85	–	–	–	–	–
28	88,357	0.031	16.0	6698.753	12.0	–	–	–	–	–
29	104,277	0.031	19.0	6699.461	14.6	–	–	–	–	–
30	123,275	0.031	22.6	6700.132	17.7	–	–	–	–	–
31	145,073	0.031	26.9	6700.784	21.4	–	–	–	–	–
32	170,409	0.022	22.6	6701.303	25.9	–	–	–	–	–
33	199,844	0.022	26.9	6701.644	31.2	–	–	–	–	–
34	235,273	0.022	26.9	6701.901	37.6	–	–	–	–	–
35	272,825	0.022	32.0	6702.117	45.7	–	–	–	–	–
36	319,389	0.022	38.1	6702.299	55.5	–	–	–	–	–
37	375,188	0.022	45.3	6702.459	67.4	–	–	–	–	–
38	443,902	0.022	53.8	6702.642	81.7	–	–	–	–	–
39	522,189	0.022	64.0	6702.815	98.8	–	–	–	–	–
40	612,931	0.022	76.1	6702.966	119	–	–	–	–	–
41	718,761	0.016	53.8	6703.061	143	–	–	–	–	–
42	844,127	0.016	64.0	6703.150	171	–	–	–	–	–
43	988,405	0.016	76.1	6703.208	205	–	–	–	–	–
44	1,149,526	0.016	90.5	6703.256	244	–	–	–	–	–
45	1,346,037	0.016	108	6703.295	291	–	–	–	–	–
46	1,583,069	0.016	128	6703.340	347	–	–	–	–	–
47	1,872,353	0.016	152	6703.381	412	–	–	–	–	–
48	2,194,659	0.011	108	6703.425	490	–	–	–	–	–
49	2,570,539	0.011	128	6703.458	580	–	–	–	–	–
50	3,008,669	0.011	152	6703.478	687	–	–	–	–	–
51	3,535,715	0.011	181	6703.498	813	–	–	–	–	–
52	4,118,331	0.011	215	6703.512	962	–	–	–	–	–

## Numerical experiment

In this section, we compute the smallest eigenvalue of (2.1) on the L-shaped domain $(0,1)^{2}\setminus[\frac{1}{2},1]^{2}$ by Algorithms 1–3 and Algorithms 1M–3M and $(0,1)^{3}\setminus ([0.5,1]\times[0,1]\times[0.5,1] )$ by Algorithms 1–2 to demonstrate the advantages of the adaptive Morley element method based on the inverse-shift iteration for a biharmonic eigenvalue problem. Our programs are compiled on MATLAB2012a under the package of Chen [28] using HP-Z230 workstation with ROM 32G and CPU 3.60 GHz.

We use the command “∖” to solve (5.2) and use the sparse solver $\operatorname{eigs}(A,B,1,{'}sm{'})$ to solve (2.3) for the smallest eigenvalues. Before showing the results, some symbols need to be explained:

$\lambda_{h_{l}}$
the smallest eigenvalue obtained by the *l*th iteration using Algorithm 1.
$\lambda^{R}_{h_{l}}$
the smallest eigenvalue obtained by the *l*th iteration using Algorithm 2.
$\lambda^{F}_{h_{l}}$
the smallest eigenvalue obtained by the *l*th iteration using Algorithm 3.
$\lambda^{M}_{h_{l}}$
the smallest eigenvalue obtained by the *l*th iteration using Algorithm 1M.
$\lambda^{RM}_{h_{l}}$
the smallest eigenvalue obtained by the *l*th iteration using Algorithm 2M.
$\lambda^{FM}_{h_{l}}$
the smallest eigenvalue obtained by the *l*th iteration using Algorithm 3M.
$N_{\mathrm{dof}}$
the number of the degree of freedom.
$\operatorname{CPU}(s)$
the time CPU runs from the first iteration to the current iteration.

**Figure 1:**
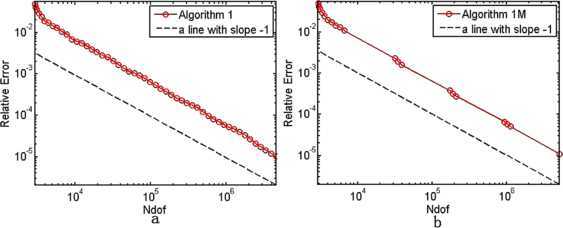
The convergence rates of the smallest eigenvalue from Algorithm 1(a) and Algorithm 1M(b)

**Figure 2:**
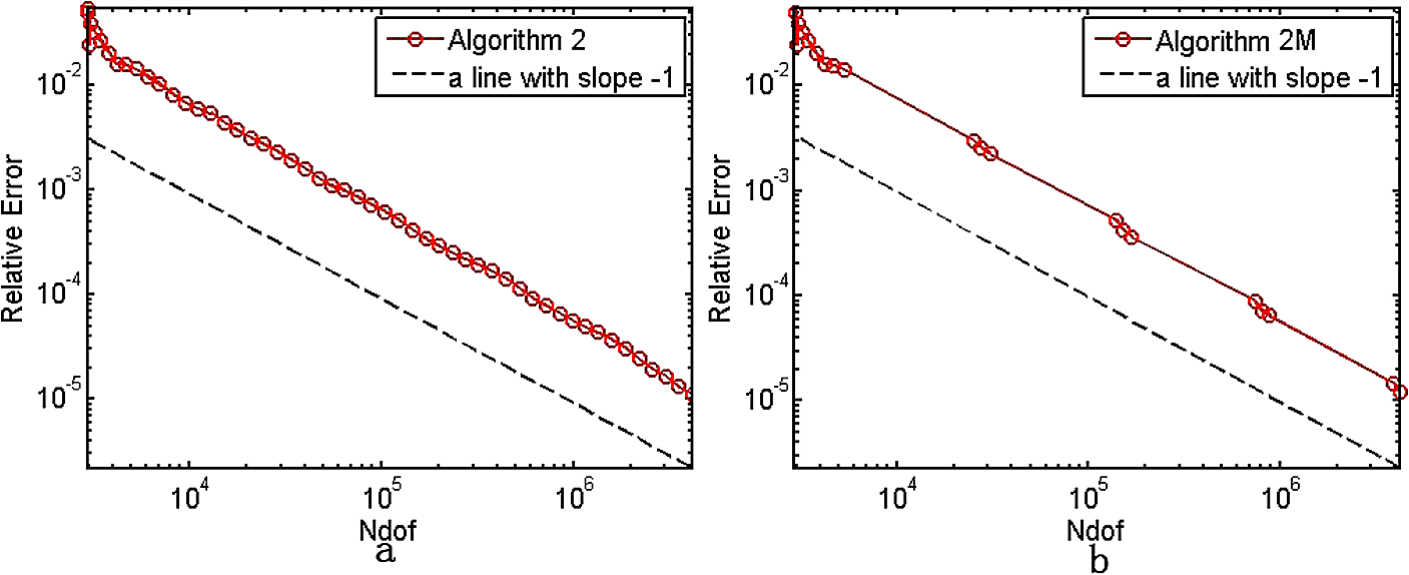
The convergence rates of the smallest eigenvalue from Algorithm 2(a) and Algorithm 2M(b)

**Figure 3:**
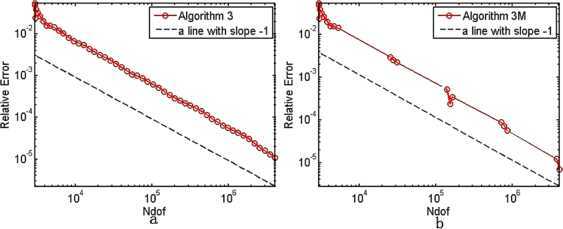
The convergence rates of the smallest eigenvalue from Algorithm 3(a) and Algorithm 3M(b)

In $\mathbb{R}^{2}$, the initial mesh $\pi_{h_{0}}$ is isosceles right triangle subdivision with mesh size $\frac{\sqrt{2}}{32}$, and we take $\theta=0.25$, $C_{r}=1.1$, $\alpha=\frac{1}{2}$. We fix shift from the 25th and 13th in Algorithm 3 and Algorithm 3M, respectively. The results are shown in Tables 1–3. We depict the error curves of Algorithms 1–3 and Algorithms 1M–3M in Figs. 1–3.

From Tables 1–3, we can get the conclusion that in the case the accurate are almost same, Algorithms 2–3 take about half time of Algorithm 1. In the case the accurate are almost same, Algorithm *i*M takes about $\frac{2}{5}$ time of Algorithm *i*, $i=1,2,3$.

The smallest eigenvalue of (2.1) is unknown. Therefore, we replace it with an approximate eigenvalue $\lambda_{1}\approx6703.585$ in $\mathbb{R}^{2}$ with high accuracy. It is present that the relative error curves of the smallest eigenvalues derived from Algorithms 1–3 and Algorithms 1M–3M on the adaptive meshes in Figs. 1–3, whose slopes are more or less −1, which shows that all the six Morley element adaptive algorithms can get the optimal convergence rate $O(h^{2})$ in $\mathbb{R}^{2}$.

**Figure 4:**
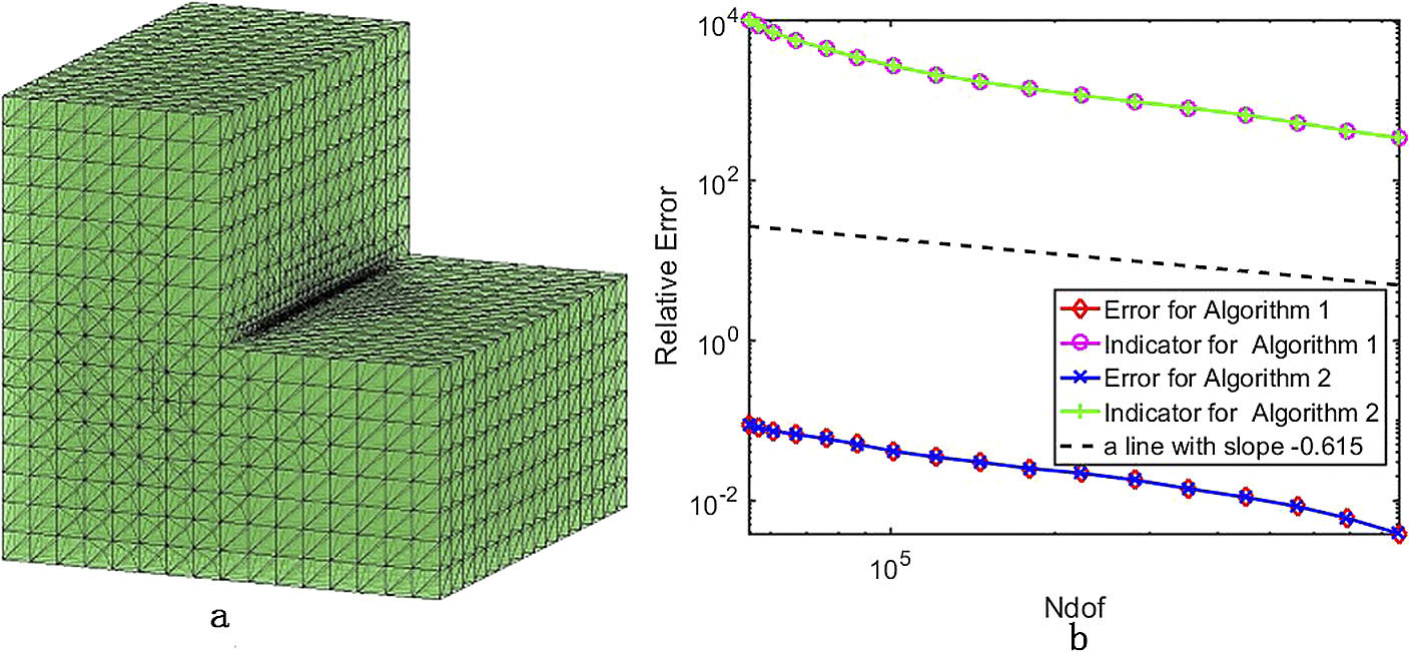
The refined mesh for the L-shaped domain (a) and the convergence rates of the smallest eigenvalue from Algorithm 1 and Algorithm 2(b) in $\mathbb{R}^{3}$

In $\mathbb{R}^{3}$, the initial mesh $\pi_{h_{0}}$ is tetrahedron subdivision with mesh size $\frac{\sqrt{3}}{16}$, and we take $\theta=0.25$ and $\lambda_{1}\approx8290.011$ with high accuracy replacing the accurate eigenvalue. It is present that the refined mesh and the relative error curves of the smallest eigenvalues derived from Algorithms 1–2 in Fig. 4, from which we see that Algorithm 2 is more efficient than Algorithm 1, but meanwhile we also see from Table 4 that the mesh size has no change. Because $N_{\mathrm{dof}}$ in $\mathbb{R}^{3}$ increases very fast after uniform refinement, which leads to surpassing computer’s memory, we cannot employ Algorithms 1M–3M to solve (2.1).

**Table 4:** The smallest eigenvalue solved by Algorithm 1 and Algorithm 2

*l*	$N_{\mathrm{dof}}$	$h_{l}$	$\lambda_{1,h_{l}}$	CPU(*s*)	$N_{\mathrm{dof}}$	$h_{l}$	$\lambda_{1,h_{l}}^{R}$	CPU(*s*)
1	54,896	0.108	7547.686	8.25	54,896	0.108	7547.686	9.71
2	57,176	0.108	7612.980	16.5	57,176	0.108	7613.142	13.0
3	60,822	0.108	7678.863	25.6	60,822	0.108	7678.902	16.7
4	67,192	0.108	7736.644	35.1	67,192	0.108	7736.699	20.9
5	76,278	0.108	7802.128	46.5	76,316	0.108	7802.062	25.8
6	86,838	0.108	7871.038	58.6	86,905	0.108	7872.137	31.2
7	101,261	0.108	7949.987	73.8	101,368	0.108	7950.349	38.4
8	121,408	0.108	8001.974	92.4	121,563	0.108	8002.931	47.3
9	146,456	0.108	8041.421	118	146,215	0.108	8040.738	58.1
10	180,528	0.108	8082.211	155	180,203	0.108	8082.247	72.3
11	224,755	0.108	8108.734	211	224,288	0.108	8108.530	92.1
12	282,583	0.108	8141.177	295	281,953	0.108	8141.009	119
13	355,133	0.108	8174.424	414	354,598	0.108	8174.437	156
14	451,162	0.108	8199.573	583	450,739	0.108	8199.502	205
15	561,904	0.108	8220.566	847	561,090	0.108	8220.433	269
16	693,222	0.108	8240.310	2368	691,963	0.108	8240.129	354
17	863,420	0.108	8258.156	9957	861,795	0.108	8258.042	469
18	–	–	–	–	1,084,848	0.108	8272.888	624
19	–	–	–	–	1,357,830	0.108	8281.060	851
20	–	–	–	–	1,730,050	0.108	8290.011	1206
